# Pressurized Solvent Extraction with Ethyl Acetate and Liquid Chromatography—Tandem Mass Spectrometry for the Analysis of Selected Conazole Fungicides in Matcha

**DOI:** 10.3390/toxics6040064

**Published:** 2018-10-25

**Authors:** Renata Raina-Fulton, Aisha A. Mohamad

**Affiliations:** Department of Chemistry & Biochemistry, Trace Analysis Facility, University of Regina; 3737 Wascana Parkway, Regina, SK S4S 0A2, Canada; aam837@uregina.ca

**Keywords:** matcha, conazole fungicides, pressurized solvent extraction, pesticide residue analysis

## Abstract

The extraction of powdered nutraceuticals is challenging due to the low water content and high concentration of matrix components that can lead to significant matrix effects in liquid chromatography-positive ion electrospray ionization-tandem mass spectrometry (LC-ESI^+^-MS/MS). In this study we assess the feasibility of using pressurized solvent extraction with ethyl acetate to reduce the co-extraction of polar matrix components. Pigment attributed to chlorophyll was removed with in-cell clean-up utilizing Anasorb 747, Florisil^®^, and C18. Visible inspection of the extracts showed that pigment was removed from matcha, a powdered green tea sample. Pressurized solvent extraction with in-cell clean-up can be utilized to remove pigments from powdered samples such as nutraceuticals. Average matrix effect of the 32 target analytes that observed mass spectrometric signal suppression or soft MS signal enhancement was −41 ± 19% with the majority of analytes having a protonated molecular ion with *m*/*z* of 250 to 412. As generally moderate signal suppression was observed for conazole fungicides and structurally related compounds analyzed by LC-ESI^+^-MS/MS, it is recommended that matrix matched or standard addition calibration is used for quantitation. Catachins, other polyphenols, and caffeine are expected to contribute to the matrix effects observed in LC-ESI^+^-MS/MS. Diniconazole, fenbuconazole, and tebufenozide were the only target analytes with severe MS signal enhancement. Low levels (0.002–0.004 mg/kg) of prothioconazole-desthio and flusilazole were detected, along with trace levels of tebuthiuron in matcha.

## 1. Introduction

Conazole fungicides are a critical group of fungicides used on a wide variety of crops including tea products. They are predominated de-methylation inhibitors and include triazoles and imidazole fungicides. Triazoles are most widely used for blister blight in teas produced in Asia. There has been a rise in commercially available powder nutraceutical products which may be consumed in multiple ways including in drinks (tea or mixed in with other drinks) or food products (e.g., baked goods and chocolate). This necessitates the need for analytical methods capable of dealing with finely powdered samples with high pigment levels.

We have previously reviewed methods for analysis of fungicides in nutraceutical products and presented some of the analytical challenges to a wide variety of fungicides currently in use [1,2]. The analysis of conazole fungicides has been accomplished by LC-MS/MS, GC-MS/MS or GC-MS methods, and is more prone than other pesticides to a variety of issues including stability issues of target analytes, pH sensitivity, carry-over problems largely attributed to strong adsorption of conazole fungicides on surfaces including tubing and other components of LC-MS/MS or GC-MS systems, and isobaric interferences in MS detection even when using tandem MS methods [1,3,4,5,6,7,8,9]. Tandem mass spectrometry provides good sensitivity and selectivity for the analysis of conazoles fungicides. Chromatographic resolution is still required due to the structural similarity and presence of isotopes for many conazoles such that isobaric interferences are common. The number of individual conazole fungicides registered for use worldwide is also increasing with small modifications to the structure such that there is a higher potential for isobaric interferences in MS detection. In addition until recently few deuterated or C-13 isotopes of conazole fungicides were commercially available such that conazoles used for medical or veterinary applications were previously utilized as internal standards [1,4]. Due to the ability of LC-MS/MS to provide short analysis times and good MS sensitivity and selectivity it is often preferred when a wide range of conazole fungicdes are analyzed. Selected conazoles may have better or similar sensitivity when GC-MS methods are used for their analysis [1,7].

Methods for the extraction and analyses of pesticides in dry nutraceutical products are more limited than other food products [2]. In addition to the very complex matrix of nutraceutical products, these products are predominantly sold in dry powder form or as tablets or capsules which is less compatible to extraction methods. There are high concentrations of pigments. Tea products such as matcha have high levels of chlorophyll, caffeine, and polyphenols [10,11,12]. Most extraction and clean-up methods for fruits and vegetables which have higher water content are based on quick, easy, cheap, effective, rugged, and safe (QuEChERS) or modified QuEChERS approaches, and when dry powders are extracted a wetting step is required for the acetonitrile salt-out extraction typically followed by filtration [7,8,9]. Sample size must be controlled for dispersive solid phase extraction (dSPE) clean-up to avoid saturation of sorbent materials. A variety of sorbents have been used for clean-up of tea matrices in dSPE or SPE including graphitized carbon black (GCB), graphite carbon/aminopropylsilanized silica gel (carbon-NH_2_), primary secondary amine (PSA) or silica, and are efficient for the removal of chlorophyll, catechins and caffeine in infused teas and other sample matrices [9,12,13,14,15]. Some methods have been able to successfully use graphitized carbon black in sample cleanup for analysis of selected conazoles, while others have found lower recoveries [8]. Planar pesticides including selected conazole fungicides are known to strongly bind to graphitized carbon black which is the most commonly used sorbent to remove pigments in extracts.

Pesticides in tea products have been predominately analyzed after infusion or a wetting step. The Japan Official Method and modified versions of this method for pesticide residues utilize a wetting step with 20 mL of water per 5 g sample for a 30-min period followed by homogenization with acetonitrile and subsequent filtration [13,14]. The salt-out acetonitrile extraction step is followed by a portion of the extract removed for subsequent clean-up. These approaches rely on good transfer rates of the fungicides from the solid matrix into water such that the fungicides must have high water solubility. Poor recoveries of <20% have been reported for conazole fungicides even with a wetting step in the extraction procedure in a variety of sample matrices including teas [14]. Desired recoveries for pesticide residue analyses methods are 85–110%. Only selected methods have completed the extraction of some pesticides or catechins in tea directly into methanol or 50/50 *v*/*v*% ethyl acetate/hexane [3,16]. Analysis of strobilurin fungicides that were extracted with ethyl acetate using pressurized solvent extraction (PSE) has been accomplished for particles collected on filters and matcha [4,17]. PSE is an alternative extraction approach to QuEChERS that allows for direct extraction of target analytes into an organic solvent without the need for the powdered sample of low water content to undergo a time-consuming wetting step or for the extract obtained to be filtered prior to clean-up. Using PSE with ethyl acetate as the extraction solvent recoveries of conazole fungicides and deuterated internal standards (diazinond_10_) are 85–110% with within batch recoveries of ±10% [4]. The objective of this study was to evaluate an adapted pressurized solvent extraction method with in-cell clean-up of matcha green tea powders targeting initial removal of pigment. We focused on the evaluation of recoveries and the matrix effects of the remaining matrix in the extract in the quantitation of a large range of conazole fungicides by LC-ESI^+^-MS/MS.

## 2. Materials and Methods

### 2.1. Materials

Ethyl acetate, acetonitrile, and methanol were of pesticide grade and supplied by Fisher Scientific. Deionized water (18 MΩ resistivity) was from a Nanopure Diamond system (Barnstead International, Dubuque, IA, USA). Aqueous solvents were passed through a 0.45 μm membrane filter from Nuclepore (Watman, Florham Park, NJ, USA). Formic acid (>88.0%) was obtained from VWR Scientific (West Chester, PA, USA). Solids or stock solutions at 100 μg/mL of pesticide standards of all test analytes were purchased from Chem Service, Inc. (West Chester, PA, USA). Solid of propiconazole-phenyld_3_ was purchased from Sigma-Aldrich (Oakville, ON, Canada). Solids of individual pesticides (~1 mg) were dissolved in 1mL of methanol with further dilution to prepare individual stock solutions at 100 μg/mL in methanol and stored at −4 °C. A further standard stock solution at 1 μg/mL of all target analytes was prepared in methanol for use in preparation of calibration standards.

Filters used for weighing the matcha solid were LABX Berkshire Engineering Clean, 10 cm diameter, and filters used in the 66 mL ASE extraction were glass fiber 934-AH^TM^, 3.0 cm diameter (VWR Scientific). Matcha is a powdered green tea product and samples were obtained from three different manufacturers that distributed product within Canada and were labeled as organic products. Anasorb 747 (40/80 mesh) was obtained from SKC Inc. (Eighty Four, PA, USA). C18 and Florisil^®^ were obtained from Sigma-Aldrich (Oakville, ON, Canada).

### 2.2. Sample Preparation

**Figure 1:**
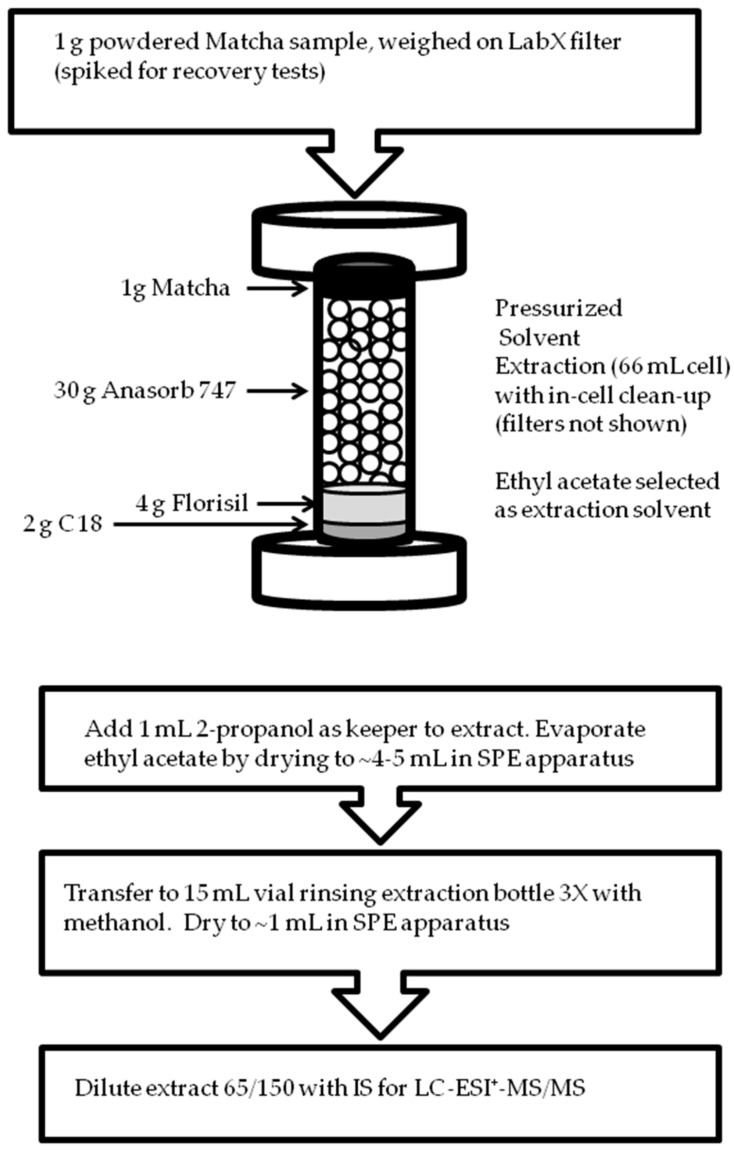
Sample preparation method for conazole fungicide analysis.

Pressurized solvent extraction with in-cell clean-up was used to extract the target analytes from the matcha samples (see Figure 1). An ASE 100 pressurized solvent extraction system (Dionex, Sunnyvale, USA) was used for extraction with the following extraction parameters: temperature 100 °C; static mode time of 30 min at 1500 psi; four static cycles; 60% flush volume; purge time with nitrogen (UHP) at the end of 600 s. The 66 mL extraction cell was loaded from bottom to top as follows: 934-AH^TM^ filter; 2 g of C18; filter; 4 g Florisil^®^; filter; 30 g Anasorb 747; two filters; 1 g of Matcha weighed in folded cleanroom grade LABX filter paper (10 cm). The LABX filter paper holding the pre-weighed matcha sample was folded to a diameter of 3 cm to fit into the extraction cell.

The extraction solvent was ethyl acetate with total volume of extraction after the four static cycles of ~130 mL. A 1 mL volume of 2-propanol was added to the extract as a keeper for the drying step. The sample extract was then dried to 4–5 mL in a SPE apparatus under slight vacuum, transferred to a 15 mL vial with methanol used to rinse the extraction bottle three times, and dried to approximately 1 mL at 0.5 mL/hr. The extract was diluted by a factor of 0.43 (65/150) with addition of internal standard (propiconazole-phenyld_3_ at 50 ng/mL) for final analysis. Standard addition calibration with internal standard was used for final analysis of the three brands of organic matcha samples. For standard addition calibrations the standard amounts added were from MDL to 80 ng/mL.

For recovery evaluation 1 g pre-weighed matcha powdered samples were spiked with 100 μL of 1 μg/mL conazole standard mix (equivalent to 0.1 mg/kg) and allowed to dry prior to loading into the extraction cell. Calibration standards (solvent-only) and matrix matched standards were prepared for evaluation of matrix effects from 0.6 to 100 ng/mL with matrix added at 1/10 dilution. The matrix was obtained from the extraction of a matcha sample with no detectable fungicides. 

### 2.3. LC-ESI^+^-MS/MS Analysis

LC analysis was performed with a Waters (Milford, MA, USA) LC system consisting of a 1525 μm binary pump and a column heater at 21 °C. A LEAP Technologies autosampler (Carrboro, NC, USA) was used for 5 μL injections at 100 μL/s and 1 pre- and post-cleans with ethyl acetate followed by methanol to minimize carry-over. A guard column (4 × 2.0 mm id, Gemini) was connected to the analytical column, Synergi Polar-RP, 550 × 2.00 mm id, 2.5 μm particle size (Phenomenex, Torrance, CA, USA). A pre-injection of 5 μL of 2-propanol was completed prior to each sample run at the initial conditions (3 v% methanol in 0.05 v% formic acid aqueous mobile phase) with a flow rate of 0.15 mL/min held for 10 min. The pre-injection of 2-propanol was completed at the initial mobile phase conditions to reduce carry-over issues. A mobile phase gradient was used for the separation of target analytes with initial conditions at 3 v% methanol in 0.05 v% formic acid aqueous mobile phase. The gradient of 0.1 v% formic acid in acetonitrile was changed linearly as follows: 0 to 1.5 min 0%; 2.5 min at 20%; 3 min at 35%; 10 min at 45%; 16 min at 50%; 18 min at 60%; 20 min at 75%; 25 min at 80% and held to 30 min. The mobile phase is re-equilibrated to 0% acetonitrile in 15 min and held for an additional 5 min prior to the pre-injection of 2-propanol. All analytes eluted in the first 25 min.

The Waters LC system was connected to a Waters Quattro Premier triple quadrupole mass spectrometer, operated in electrospray positive ion mode. The temperature of the source was set to 120 °C, desolvation temperature to 350 °C, desolvation gas flow was 750 L/h, and cone gas flow was 150 L/h. The optimized settings for ESI^+^ were capillary voltage of 3.1 kV; and Rf lens, 0.1 V. The collision gas used for SRM was argon (UHP) at 0.15 mL/min or 4 × 10^−4^ mbar. Cone voltage and collision energy were set up in the MS method as previously report [4], and shown in Table 1 for target analytes. Infusion experiments were conducted for each individual target analyte to determine the SRM conditions with a syringe pump flow rate of 50 μL/min. Table S1 (see Supplementary Material) shows the regression coefficient of the matrix matched calibration curve for each analyte at the quantitative SRM. The ratio of response for SRM1/SRM2 are within relative standard deviation criteria of <20% for all target analytes.

## 3. Results

### 3.1. Cell Design for Pressurized Solvent Extraction of Matcha

Matcha was used as a model case for analysis of conazole fungicides and structurally related pesticides in powdered samples with high levels of pigments in the sample matrix. Potential interferences in MS detection from the matrix of matcha samples includes chlorophyll, caffeine, catechins and other polyphenols which if present in samples injected would co-elute in a reversed-phase LC separation [10,11,13,14,16]. These matrix components can lead to interferences in MS detection and signal suppression or enhancement in MS detection. Powdered samples may require additional contact time with solvents to ensure complete extraction based on our prior work on extraction of fungicides from particles collected on glass fiber filters [17]. The pressurized solvent extraction procedure with in-cell clean-up was designed to firstly remove the pigment attributed to chlorophyll from the sample. The sample was loaded at the top of the extraction cell held in LABX filter paper for easier removal of the solid after extraction. Two glass fiber filters (3 cm diameter) were placed between the sample and Anasorb 747 sorbent to remove residue solids that pass through the LABX filter paper holding the matcha powdered sample. Ethyl acetate was selected as it provides good recoveries for conazole fungicides from other solid materials and the use of a less polar solvent than acetonitrile, acetonitrile/water or methanol reduces co-extraction of more polar matrix components that can lead to suppression of MS signal and interferences in MS detection. Pressured solvent extraction with ethyl acetate has been used in the extraction of catechins from teas including matcha with lower levels of caffeine found in the extracts [16]. PSE with ethyl acetate or liquid–liquid extractions have also been used to extract selected conazole and strobilurin fungicides from other teas, and solid sorbents used in air sampling [2,3,4]. Ethyl acetate has also been used with QuEChERS rather than acetonitrile for extraction of fungicides from fruit and vegetable matrices [9]. To reduce co-extraction of less polar matrix components such as fat soluble co-extracts more polar solvents such as acetonitrile or acetone are selected for extraction and have been used to extract fungicides from soil, plant and animal based foods [18,19].

The extraction cell size (66 mL) was selected to enable 30 g of Anasorb 747 to be loaded in the cell (directly below the sample) and this amount of sorbent was required to remove the green pigment coloration of the extracts. Without the use of Anasorb 747 extracts were too high in chlorophyll content such that after preconcentrated (drying) step there was precipitation. To remove the residual color in the ethyl acetate extracts 4 g of Florisil^®^ was required which was placed in the extraction cell below the Anasorb 747. We choose to also add 2 g C18 to aid in removal of some potential matrix components including pigment. Conazole fungicides recoveries have been shown to be good with both C18 and Florisil^®^ SPE clean-up [3,4,13,14,15]. Filters were placed in the extraction cell before and after each sorbent layer to allow the sorbents to be removed separately. Visible inspection of the sorbent materials after extraction showed that Florisil^®^ and to a lesser extent C18 were removing the residue pigment after the solvent had passed through the Anasorb 747. Graphitized carbon black and primary secondary amine (PSA) were also tested but were not used as the extracts obtained still had green coloration after the pressurized solvent extraction step. PSA has been found to work less effectively for clean-up in the presence of high pigment levels [15]. The use of Anasorb 747 over graphitized carbon black for pressurized solvent extraction was preferred for improved flow characteristics (larger particle size (20/40 mesh) and was the most efficient sorbent tested at removing most of the color of the extracts. Other carbon based sorbents that have been used in the literature include Carbon-X in a SPE format for green tea supplements [15]. Based on our prior procedure where we extracted fungicides from particles on filters we selected four static stages followed by a 60% flush after each static stage to ensure adequate contact time of the solvent with the finely powdered matcha sample. Fungicides are known to strong bind to many solid sorbent materials. An addition extraction with 4 static stages showed no visible color in the extract and also no presence of pesticides.

### 3.2. Modifications to the LC-ESI^+^-MS/MS Analysis

We modified our existing LC-ESI^+^-MS/MS method to include some new fungicides or additional structurally related pesticides that have become commercially available since our initial development (see Table 1) [4]. Sulfentrazone, which is a triazolone herbicide, has few existing methods [5,6]. We also re-optimized the separation for shorter total analysis time from ~45 min to 25 min with the addition of a small percentage of methanol rather than 5% 2-propanol in the aqueous mobile phase. In the prior method 2-propanol was added in the aqueous mobile phase to reduce carry-over issues, however, additional of methanol to the aqueous mobile phase rather than 2-propanol results in better chromatographic resolution for isobaric compounds. To avoid issues with carry-over from sorption of matrix or conazole fungicides in the LC-MS/MS system we completed a pre-injection of 2-propanol prior to each run for 10 min at initial mobile phase conditions. 

**Figure 2:**
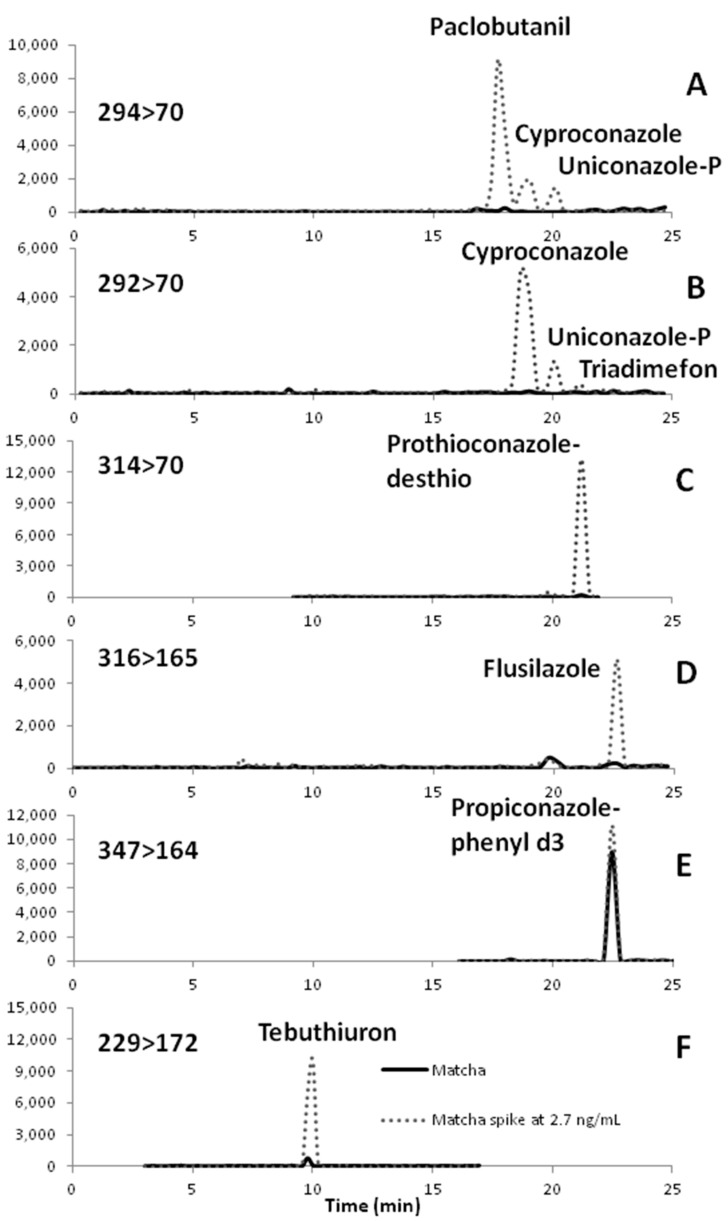
Selected Reaction Monitoring (SRM) Chromatograms of Matcha and Matcha Spiked with Standard Solution. Sample size of 1 g of Matcha with pre-concentrated of pressurized solvent extraction extract to a volume of 1 mL with extract diluted by factor of 0.43 with methanol. Sample extract is spiked at level of 2.67 ng/mL of standard mixture of conazole fungicides and structurally similar pesticides. Internal standard (propiconazole-phenyld_3_) added to sample at concentration of 50 ng/mL. SRM Chromatograms: A, SRM 294>70; B, SRM 292>70; C, SRM 314>70; D, SRM 316>165; E, SRM 347>164; F, SRM 229>72. See Table 1 for retention times of analytes.

Good chromatographic resolution was obtained for all compounds with the same or similar SRMs. The conazole fungicides shown in Figure 2A,B have similar molecular weight range are chlorinated and produce the *m*/*z* = 70 fragment that is attributed to the triazole moiety. Good separation of these compounds and retention time stability must be obtained to avoid false positive identification as particularly cyproconazole and uniconazole-P give response at SRM of 292>70 and 294>70 (quantification and confirmation SRMs). Co-elution of these fungicides with isobaric interferences occurs and worsens with matrix issues [1,4,17,20]. Similarly hexaconazole and prothionconazole-desthio elute in a similar retention time range and give response at 314>70 (Figure 2C) as well as 312>70 and 316>70. Prothioconazole-desthio has a significantly higher abundance than hexaconazole at 314>70. A small amount of 2-propanol could be added to the aqueous mobile phase to further reduce potential sorption of matrix or conazoles in the LC-MS/MS system with a recommendation to kept the percentage below 2% if methanol is also used in the aqueous mobile phase to ensure adequate chromatographic resolution of isobaric fungicides without the need for long analysis times. When matrix or conazole sorption occurs in the system this is usually evident by a shift to longer retention times and broadening of peak shapes.

### 3.3. Method Detection Limits and Calibration

Table 1 shows the quantitative and confirmation SRM for all target analytes. All new conazole or structurally similar analytes (imazamox, tricyclazole, sulfentrazone, etaconazole, etoxazole) were separated from each other. Etaconazole at higher concentrations can give a response at 328→70, and 326→70, however the response of diniconazole is much stronger at these SRMs and the two conazoles are separated. Table 1 shows the method detection limits (MDLs) as determined by the lowest concentrations of standard (matrix matched) that deviate from the regression line by <25%. The method detection limits for 29 of the 35 analytes were slightly higher than we previously reported with solvent-based standards [4], but still in a desirable range of 0.0006–0.010 mg/kg. The new target analytes (imazamox, sulfentrazone, and etacoanzole) had detection limits of 0.010 mg/kg. Method detection limit for etaconazole and tricyclazone were lower at 0.001 and 0.002 mg/kg, respectively. The ratio of SRM1/SRM2 were determined on the same day of analysis from matrix matched standards, with relative standard deviation <20% (see Supplementary Material Table S1). Our working calibration range was MDL to 0.080 mg/kg. Correlation coefficient of calibration curves obtained from matrix matched standards was >0.94 (see Table S1). Tetraconazole observed a lower *r*^2^ (0.91) which was attributed to severe matrix effects.

### 3.4. Matrix Effects and Recoveries of Pressurized Solvent Extraction

To evaluate the extent of matrix suppression or enhancement on MS signal in LC-ESI^+^-MS/MS the slope of best fit line obtained with calibration standards of the same concentration in calibration standards with matrix added (m_matrix_) and those obtained with solvent-only (m_solvent_) were compared. The matrix effect (ME) was calculated using the following formula:

(1)  ME = ((mmatrix−msolvent)−1)*100% 

The matrix was obtained from an extract of a matcha powder sample which was shown to have no target analytes detectable (signal/noise ratio < 3). Most fungicides observed soft (+20 to −20%) or moderate matrix effects (±20–50%). Signal suppression rather than enhancement in MS detection was more commonly observed after removal of the chlorophyll from the sample extract. Average matrix effect of 32 target analytes with suppression or soft signal enhancement was −41 ± 19%. The majority of these target analytes have a mass to charge of 250 to 412 for the protonated molecular ion. Diniconazole, fenbuconazole, and tebufenozide were the only analytes with severe MS signal enhancement (102%, 81%, 225%, respectively). A larger number of analytes (6 of 8) with severe signal suppression (range −55% to −75%) eluted between 20–25 min in the separation. Febuconazole, diniconazole, and difenconazole also observed shifts in the baseline (see Figure S1) and had low recoveries (see Table 2). For conazole fungicides matrix matched standards or standard addition calibration and use of an internal standard are necessary to provide reliable quantitation. Addition of higher amounts of matrix than 50% of solvent composition of injected sample lead to instability of retention times such that the samples for subsequent analysis were diluted generally by a factor of 0.43 (65/150) with methanol. 

Table 2 shows that acceptable recoveries (70–120%) were obtained for selected conazole fungicides including tebuthiuron, triadimenol, myclobutanil, and triadimenfon. The recovery of prothioconazole-desthio which was detected in one of the matcha samples was 69% with a relative standard deviation >20%. Cyproconazole and paclobutanil also had recoveries in the 50–70% range (see Table 2). While these recoveries are not ideal they are still an improvement over methods reported for this difficult sample matrix [2]. Other less commonly analyzed conazoles observed low recoveries (not listed in Table 2 due to recoveries <20%), although MS signal suppression was moderate indicating that either these fungicides were strongly adsorbed on Anasorb 747 or not adequately removed from the powdered matcha sample. Additional extraction with ethyl acetate did not show any detectable levels of fungicides.

For subsequent analysis of matcha product samples (labeled as organic) from three different manufacturers standard addition calibration was utilized with propiconazole-phenyld_3_ for the internal standard. Target analytes that were at or near our method detection limits included tebuthiuron, prothioconazole-dethio, and flusilazole. Figure 2C–E shows the SRM chromatograms for sample injection with and without standard addition for prothioconazole-desthio, flusilazole, and tebuthiuron. Two of the three commercial products did not contain any target analytes above the method detection limit. Tebuthiuron levels were just below our MDL for the injected sample at a dilution factor of 0.43 (see Figure 2). With improved extraction and clean-up the analysis of these target analytes would be more reliable and based on the current method detectable amounts are in the range of 2–4 μg/kg which is comparable to other reports of conazole fungicides in green tea leaves [2,21]. There have been no prior reports of detectable levels of conazole fungicides or tebuthiuron in matcha.

## 4. Discussion

An additional clean-up step following the pressurized solvent extraction with in-cell clean-up was not used. SPE clean-up following extraction and pre-concentration of the extract is a commonly used approach to further remove matrix. Given the sorbents used in the in-cell clean-up the most common alternative sorbent for SPE clean of extracts would be silica to remove residual caffeine in the ethyl acetate extracts, however it has already been reported that a large number of conazole fungicides have poor recoveries with this sorbent material [13,15]. Filtration with polyvinyldifluoride membrane filters has been shown to reduce epicatechin and epigallocatechin gallate concentrations in acetonitrile extracts [10], and this could be further evaluated for recoveries of conazole fungicides in different solvents used for pressurized solvent extraction.

Ethyl acetate is a less polar solvent than typically used in modified QuEChERS methods, Official Japan method, or modification of this method that involve a wetting step of the solid followed by an acetonitrile salt-out extraction. It has been shown that ethyl acetate extracts obtained with pressurized solvent extraction have lower caffeine content but high concentrations of catechins and polyphenols [16]. Other options for further removal of polyphenols without removal of conazole fungicides includes the use of other non-carbon based sorbents such as polymeric resins or green extraction resins such as chotisan which still need to be evaluated for application to extraction of nutraceuticals [21]. Including a more nonpolar solvent such as hexane or cyclohexane with ethyl acetate as an extraction solvent mixture for pressurized solvent extraction may also further reduce the presence of catechins and other polyphenols in the extract solvent [3,19]. Difenoconazole has been shown to be efficiently extracted with both ethyl acetate and 50/50 *v*/*v* mixtures of ethyl acetate and hexane from chrysanthemum flower tea [3]. Poor recoveries of difenconazole and matrix effects in our study indicate that both MS interferences and strong sorption onto sorbents used for clean-up are the most likely sources of low recoveries of conazole fungicides. Poor extraction recoveries <20% were observed for all target analytes not reported in Table 2 with the potential source including strong adsorption onto the Anasorb 747 (a carbon based material) or poor contact of the solvent with the powdered sample which may also result in larger (>20%) relative standard deviation of recoveries for some conazole fungicides. MS signal suppression was moderate for most analytes, and matrix matched calibration curves in the desired calibration range from MDL-0.080 mg/kg had correlation coefficients >0.94 (see Table S1).

The standard deviation of the recoveries of conazole fungicides from matcha were also larger for some fungicides (>20%) with recoveries in the range of 60–70% or lower. There were also noticeable differences in matrix interferences in repeated extractions of the same manufacturer’s sample of matcha as seen in baseline shifts in some SRM chromatograms. Lower extraction recoveries for biteranol, cyproconazole, simeconazole, and triadimenol for the matcha samples relative to other tea samples have also been reported [14]. To obtain relative standard deviation for recoveries in the desired range (<20%) for all target analytes it may be necessary to increase the contact of the solvent with the fine powdered sample by mixing the matcha with a sorbent such as a polymeric resin or Florisil^®^. This may also allow us to reduce the amount or need for Anasorb 747 for removal of chlorophyll in the extracted solvent.

## 5. Conclusions

The clean-up of extracts from powdered nutraceutical samples is a challenge. Extracts of matcha infused in water have inheritably better relative standard deviation of recoveries of selected conazoles as only the water soluble components are extracted, however other conazoles still show poor recoveries [14]. Evaluation of a wider range of conazole fungicides and other structurally similar pesticides showed that there are similar issues when using PSE with poor extraction recoveries for the less commonly analyzed conazole fungicides. In general matrix suppression of the MS signal was moderate indicating a need for matrix matched or standard addition calibration. PSE with in-cell clean-up can be used to remove pigments from powdered nutraceutical products such as matcha. There are more commercially available products with high pigment levels such that new extraction and clean-up methods need to be developed. This analysis shows that conazole fungicides strongly adsorb to many of the sorbent materials used for pigment removal such that alternate sorbents from carbon based materials need to be evaluated in the future. Matcha is a very difficult sample matrix and selected conazole fungicides can be extracted directly into ethyl acetate with subsequent in-cell clean-up to remove chlorophyll. The first detection of flusilazole, prothioconazole-desthio and trace levels of tebuthiuron are presented. The MS signal suppression that is still present is attributed to the presence of polyphenols including catechins that future extraction and clean-up methods need to address. This work shows that issues with MS signal suppression are also caused by other components of matcha than pigment. Further reducing the polarity of the extraction solvent while maintaining good recoveries for conazole fungicides, and clean-up with Florisil^®^, polymeric or alternative green sorbent materials should be evaluated. Of the carbon based sorbents and other sorbents evaluated Anasorb 747 was best able to remove high amounts of the green pigment from ethyl acetate extracts such that filtration was not required after the pre-concentration of extracts. Anasorb 747 also has potential for re-use after clean-up to reduce sample analysis costs. To improve the method development strategy utilizing green analytical approaches, improvements in PSE with in-cell clean-up are needed to deal with difficult sample matrices without the need for subsequent off-line clean-up steps [22,23]. To further reduce matrix effects caused by more polar matrix components, a membrane filter could be tested for use with PSE in-cell clean-up. Some membrane filters strongly absorb polyphenols, and this may aid in reducing matrix issues. Pressurized solvent extraction is well suited to address filtration in the extraction cell as extracts are filtered as the solvent is pushed out of the extraction cell with nitrogen gas.

## Figures and Tables

**Table 1:** Selected Reaction Monitoring Transitions (SRMs) for Target Analytes Analyzed by LC-ESI^+^-MS/MS.

Target Analyte	Retention Time (min)	Quantitative SRM, Confirmation SRM (Cone Voltage, Collision Energy)	Method Detection Limits with Matrix Matched Standards (mg/kg)
Benzotriazole	8.24	120→65 (40,17), 120→92 (40,17)	0.0006
Sulfathiazole	8.46	256→155 (20,15), 256→92 (20,25)	0.002
Imazamox	9.09	306→261 (40,20), 306→217 (40,20)	0.010
Sulfamethizole	9.34	271→156 (20,15), 271→92 (20,25)	0.002
Tebuthiuron (thiadiazolylurea herbicide)	10.48	229→172 (25,15), 229→116 (25,25)	0.001
Tricyclazole (benzothiazole fungicide)	12.06	190→163 (35,20), 190→136 (35,25)	0.002
Sulfentrazone	15.36	387→307 (35,20), 389→309 (35,20)	0.010
Imazalil	15.8	297→159 (20,25), 297→201 (20,20)	0.010
Thioconazole	15.93	391→130 (20,20), 391→360 (20,10)	0.010
Azaconazole	16.71	300→159 (30,25), 300→231 (30,15)	0.001
Triadimenol	18.19	296→70 (15,15), 298→70 (15,15), 296→99 (15,10)	0.002
Paclobutrazol (plant growth regulator with triazole moiety)	18.58	294→70 (30,20), 295→70 (25,20), 296→70 (15,15)	0.010
Triticonazole	19.39	318→70 (20,15), 320→70 (20,20)	0.010
Cyproconazole	19.76	292→70 (30,15), 294→70(30,20)	0.002
Hexaconazole	20.58	314→70 (25,20), 316→70(25,20)	0.010
Uniconazole (uniconazole-P)	20.94	292→70 (30,15), 294→70 (30,20)	0.010
Etaconazole	21.58	328→159 (30,25), 330→161 (30,25), 328→187 (30,30)	0.001
Prochloraz	21.61	376→70 (15,25),378→70 (15,25), 376→308 (15,15)	0.010
Myclobutanil	21.73	289→70 (25,15), 291→70 (25,15)	0.010
Triadimefon	21.73	295→70 (25,20), 297→70 (25,20)	0.002
Prothioconazole (analyzed as prothioconazole-desthio)	21.75	314→70 (25,20), 312→70 (25,20), 312→125 (25,20)	0.005
Tebuconazole	21.94	308.5→70 (35,20), 310.5→70 (35,20), 308.5→125 (35,20)	0.001
Bromuconazole	22.01	376→159 (30,25), 378→159 (30,25)	0.010
Penconazole	22.12	284→70 (25,15), 284→159 (25,15)	0.010
Metconazole	22.15	321→70 (30,20), 323→70 (30,20)	0.010
Diniconazole	22.46	326→70(35,25), 328→70(35,25), 326→159 (35,20)	0.0006
Epoxiconazole	22.46	330→121 (25,20), 332→121 (25,20), 330→123 (25,20)	0.010
Tetraconazole	22.46	372→159 (30,25), 372→70 (30,25)	0.010
Biteranol	22.73	338→99 (15,15), 338→269 (15,15)	0.002
Propiconazole	22.73	342→159 (30,25), 342→69 (30,25)	0.010
Flusilazole	22.94	316→165 (30,25), 316→248 (30,15)	0.0006
Fenbuconazole	23.12	337→70 (30,20), 337→125 (30,20)	0.001
Tebufenozide (insecticide)	23.12	353→133 (12,17), 353→297 (12,17)	0.002
Difenoconazole	23.64	406→251 (30,25), 408→253 (30,25)	0.010
Etoxazole	25.02	360→57 (35,25), 360→141 (35,30), 360→177.5 (35,20)	0.010
Propiconazole-phenyld_3_ (internal standard)	22.80	347→164 (50,25), 349→166 (50,25), 347→69 (50,25), 349→69 (50,25)	NA

**Table 2:** Matrix Effect and Detected Concentration of Selected Fungicides in Matcha.

Target Analyte (SRM)	Recovery, Spiked at 0.01 mg/kg ^1^ (Average ±SD, *n* = 4 )	% Matrix Effect	Detected Concentration in Matcha (mg/kg) ^2^
*Selected Analytes with MS Signal Suppression or Soft Enhancement*			
Tebuthiuron (229→172)	80.7 ± 4.70	−19%	ND
Sulfentrazone (387→307)	64.0 ± 18.3	−35%	ND
Triadimenol (296→70)	109.5 ± 11.7	−32%	ND
Paclobutanil (295→70)	51.8 ± 14.0	−38%	ND
Cyproconazole (292→70)	69.3 ± 12.9	−37%	ND
Uniconazole (292→70)	23.9 ± 12.9	−29%	ND
Myclobutanil (291→70)	84.9 ± 38.3	−53%	ND
Triadimenfon (295→70)	96.1 ± 34.6	−44%	ND
Hexaconazole (314→70)	12.1 ± 19.8	−75%	ND
Prothioconazole-desthio (314→70)	69.2 ± 29.2	−54%	0.0035
Flusilazole (316→165)	40.4 ± 28.9	6%	0.0024
Propiconazole (342→159)	41.9 ± 25.0	−54%	ND
Etaconazole (330→161)	49.1 ± 11.3	−5%	ND
Azaconazole (300→159)	32.3 ± 11.0	−33%	ND
Difenconazole (406→251)	20.4 ± 87.9	−47%	ND
*Analytes with Severe Signal Enhancement*			
Diniconazole (326→70)	21.5 ± 5.40	102%	ND
Fenbuconazole (337→70)	29.9 ± 8.10	81%	ND

^1^ Matrix Matched Standards; ^2^ Standard addition calibration; ND (not detected) < MDL.

## Supplementary Materials

The following are available online at http://www.mdpi.com/2305-6304/6/4/64/s1, Figure S1: Selected Reaction Monitoring Chromatograms of Diniconazole With and Without Matrix Added, Table S1: Method Detection Limit, Regression Coefficient of Matrix Matched Calibration Curve, and Percentage Matrix Effects for Target Analytes Analyzed by LC-ESI^+^-MS/MS.
