# Supplementary material for: Pressurized Solvent Extraction with Ethyl Acetate and Liquid Chromatography—Tandem Mass Spectrometry for the Analysis of Selected Conazole Fungicides in Matcha
Source: Toxics. 2018 Oct 25;6(4):64. doi: 10.3390/toxics6040064 (PMC6315461; doi:10.3390/toxics6040064)
Supplement: Supplementary file 1 [file toxics-06-00064-s001.pdf]

Supplementary Material: Pressurized Solvent Extraction with Ethyl Acetate and Liquid Chromatography –Tandem Mass Spectrometry for the Analysis of Selected Conazole Fungicides in Matcha

Renata Raina-Fulton and Aisha A. Mohamad

Table S1. Method Detection Limit, Regression Coefficient of Matrix Matched Calibration Curve, and Percentage Matrix Effects for Target Analytes Analyzed by LC-ESI+-MS/MS.

Target Analyte	Retention time (min)	% Matrix effects	MDL with matrix matched standards (mg/kg)	R^2	Ratio Response SRM1/SRM2±RSD
Benzotriazole (120→65)	8.24	−32	0.0006	0.962	2.84 ± 0.6
Sulfathiazole (256→155)	8.46	−66	0.002	0.988	1.87 ± 6.3
Imazamox (306→261)	9.09	−32	0.010	0.984	3.86 ± 12.9
Sulfamethizole (271→156)	9.34	−63	0.002	0.987	1.92 ± 10.0
Tebuthiuron (229→172)	10.48	−19	0.001	0.966	3.41 ± 8.4
Tricyclazole (190→63)	12.06	−23	0.002	0.967	1.22 ± 4.7
Sulfentrazone (387→307)	15.36	−35	0.010	0.970	1.43 ± 18.3
Imazalil (297→159)	15.8	−33	0.010	0.970	2.04 ± 12.6
Thioconazole (391→130)	15.93	−35	0.010	0.963	92.9 ± 18.4
Azaconazole (300→159)	16.71	−33	0.001	0.969	2.05 ± 2.9
Triadimenol (296→70)	18.19	−32	0.002	0.980	3.61 ± 8.9
Paclobutrazol (294→70)	18.58	−38	0.010	0.961	10.3 ± 12.7
Triticonazole (318→70)	19.39	−33	0.010	0.961	3.04 ± 2.2
Cyproconazole (292→70)	19.76	−37	0.002	0.988	5.02 ± 12.5
Hexaconazole (314→70)	20.58	−75	0.010	0.979	5.51 ± 14.7
Uniconazole (uniconazole-P) (292→70)	20.94	−29	0.010	0.971	0.74 ± 14.6
Etaconazole (330→161)	21.58	−5	0.001	0.997	10.0 ± 13.6
Prochloraz (376→70)	21.61	−42	0.010	0.978	1.16 ± 10.9
Myclobutanil (289→70)	21.73	−53	0.010	0.996	2.95 ± 11.2
Triadimefon (295→70)	21.73	−44	0.002	0.949	3.14 ± 14.6
Prothioconazole (analyzed as prothioconazole-desthio) (314→70)	21.75	−54	0.010	0.972	8.59 ± 16.7
Tebuconazole (308→70)	21.94	−7	0.001	0.996	3.10 ± 7.4
Bromuconazole (376→159)	22.01	−66	0.010	0.989	0.99 ± 6.0
Penconazole (284→70)	22.12	−67	0.010	0.993	7.29 ± 6.8
Metconazole (321→70)	22.15	−61	0.010	0.974	3.61 ± 18.9
Diniconazole (326→70)	22.46	102	0.0006	0.997	1.53 ± 8.7

Epoxiconazole (330→121)	22.46	−31	0.010	0.974	3.20 ± 8.2
Tetraconazole (372→159)	22.46	−75	0.010	0.913	1.39 ± 5.9
Biteranol (338→99)	22.73	−43	0.002	0.997	7.32 ± 17.0
Propiconazole (342→159)	22.73	−54	0.010	0.986	0.91 ± 11.9
Flusilazole (316→165)	22.94	6	0.0006	0.997	1.71 ± 9.8
Fenbuconazole (337→70)	23.12	81	0.001	0.999	2.02 ± 18.5
Tebufenozide (353→133)	23.12	225	0.002	0.997	36.2 ± 9.3
Difenoconazole (406→ 251)	23.64	−47	0.010	0.981	1.45 ± 2.1
Etoxazole (360→57)	25.02	−27	0.010	0.946	4.34 ± 8.4

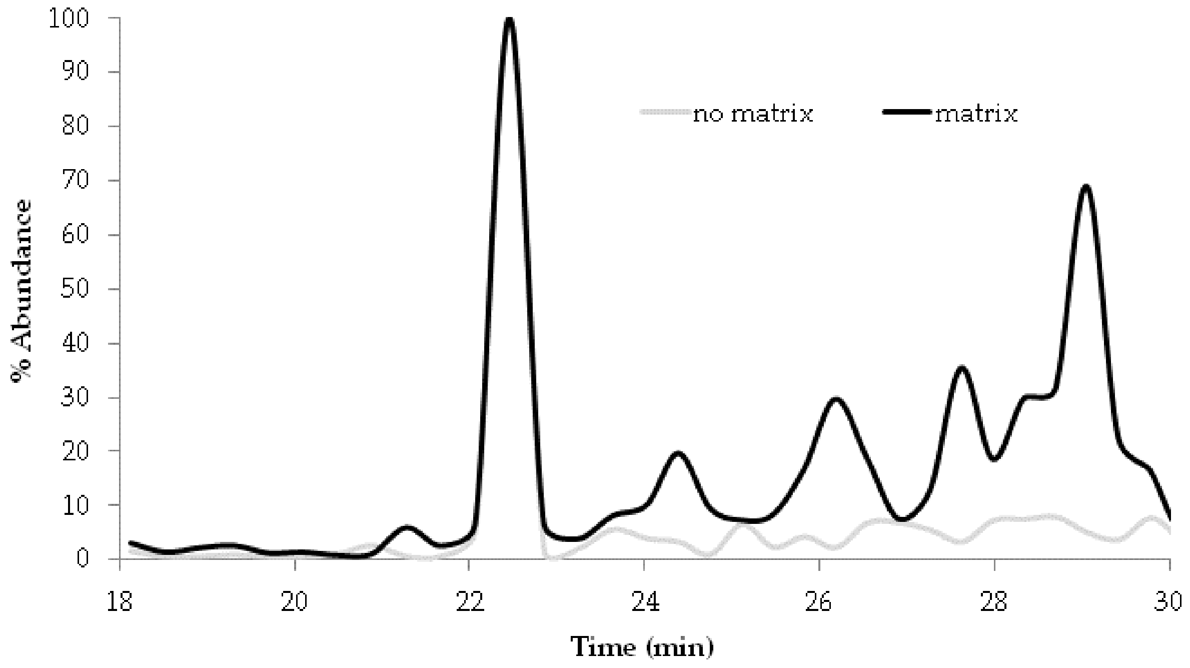

Figure S1. Selected reaction monitoring (327→70) chromatograms of diniconazole standard (0.001 mg/kg) with and without matrix added. Retention time of diniconazole is 22.5 min.
